# Long-Term Weight Change: Association with Impaired Glucose Metabolism in Young Austrian Adults

**DOI:** 10.1371/journal.pone.0127186

**Published:** 2015-05-29

**Authors:** Katharina Wirth, Raphael S. Peter, Christoph H. Saely, Hans Concin, Gabriele Nagel

**Affiliations:** 1 Institute of Epidemiology and Medical Biometry, Ulm University, Ulm, Germany; 2 Vorarlberg Institute for Vascular Investigation and Treatment (VIVIT), Feldkirch, Austria; 3 Department of Medicine and Cardiology, Academic Teaching Hospital Feldkirch, Feldkirch, Austria; 4 Private University of the Principality of Liechtenstein, Triesen, Liechtenstein; 5 Agency for Preventive and Social Medicine, Bregenz, Austria; University College London, UNITED KINGDOM

## Abstract

Little is known about the associations between long-term weight change and the natural history of impaired fasting glucose (IFG) in young adults. We investigated the association between long-term body mass index (BMI) change and the risk of IFG using data of 24,930 20- to 40-year-old participants from the Vorarlberg Health Monitoring and Promotion Program (VHM&PP) cohort. Poisson models were applied to estimate the 10-year risk for new development of IFG (≥5.6 mmol/L), and persistence of IFG. Over 10 years, most men (68.2%) and women (70.0%) stayed within their initial BMI category. The risk for incident IFG was highest for men and women with persisting obesity (37.4% and 24.1%) and lowest with persisting normal weight (15.7% and 9.3%). Men transitioning from normal to overweight increased their risk of incident IFG by factor 1.45 (95%-CI: 1.31, 1.62), women by 1.70 (95%-CI: 1.50, 1.93), whereas transitioning from overweight to normal weight decreased the risk in men by 0.69 (95%-CI: 0.53, 0.90) and 0.94 (95%-CI: 0.66, 1.33) in women. Relative risks for men and women transitioning from obesity to overweight were 0.58 and 0.44, respectively. In conclusion, 10 year weight increase was associated with an increased IFG risk, weight decrease with a decreased risk of IFG in young adults.

## Introduction

In Austria the prevalence of overweight or obesity among adults is high (male: 50.0%, female: 39.6%) and has been increasing during the past decades [1]. A recent publication of the Health-AARP (formerly the American Association of Retired Persons) Diet and Health Study revealed that subjects were lean until the age of 18 years, but gained considerable weight until the age of 50 years [2]. Recent reports showed an increase of adiposity prevalence in young adults in Germany and Austria [3,4]. In the Vorarlberg Health Monitoring and Promotion Program (VHM&PP) cohort strongest weight gain was observed in men aged 20 to 40 years [5]. Obesity is associated with adverse metabolic changes [6,7]. In particular, there is evidence that an early onset of overweight or obesity is associated with an augmented risk for developing the metabolic syndrome [7], type two diabetes mellitus (T2DM) [8] and vascular disease [9]. There is evidence that the age of onset of T2DM is falling [10] and that the risk due to obesity in young adults persists independently of later weight changes [11,12].

Impaired fasting glucose (IFG) predicts T2DM, with about 25% of those affected progressing to T2DM within a period of three to five years [13,14]. Also, longitudinal studies indicate that IFG itself is associated with modest increase in the risk for cardiovascular events [15].

Little is also known about the prevalence and the natural history of IFG. In terms of: How likely are normoglycemic individuals to progress to IFG? What proportion of individuals with IFG stay in the IFG state or revert to normoglycemia over time? And which modifiable risk factors can effectively influence the natural history of IFG? Unfortunately most studies on the IFG prevalence or the natural history of IFG are based on the older cut point of 6.1 mmol/L (110 mg/dL) and not on current criteria for the diagnosis of IFG [13,14].

Understanding the relationship between weight change and glycemic dysregulation in young adults is increasingly important given the rapid increase in the prevalence of obesity in this age group. Thus, the aim of our study was to investigate the associations between long-term weight change and the natural history of IFG in young adults based on the current cut point of 5.6 mmol/L (100 mg/dL).

Further, weight change may have different implications depending on the baseline BMI of a person. Therefore we wanted to display the interactive relationship between BMI at baseline and BMI change on risk of IFG development and persistence using generalized additive models (GAM). GAM allowed estimating the IFG risk for specific combinations of baseline BMI and BMI change without making assumptions about the form of the association with IFG.

## Materials and Methods

### Study population and design

The VHM&PP is a population-based risk factor surveillance program in Vorarlberg, the westernmost province of Austria. The program is administrated by the Agency of Social and Preventive Medicine. All adults (aged ≥ 19 years) within the province were invited by letter, newspaper, radio and television to participate. Between January 1985 and June 2005, 185,316 adult Vorarlberg residents (53.9% female) were enrolled in the VHM&PP study cohort after written informed consent. Participants were mainly Caucasians. Pregnant women were not included in the program. Most of the participants had two or more registered visits with varying time intervals between them. Details of the program and characteristics of the study population have been described previously [16,17].

The program includes a physical examination with measuring of systolic and diastolic blood pressure, height, weight, total cholesterol, triglycerides, γ-glutamyltransferase, and plasma glucose. since January 1988 samples were taken after an overnight fast. The screening examinations took place in the practices of local physicians according to a standard protocol. Anthropometric measures were carried out by medical staff with participants wearing light indoor clothes and no shoes. Smoking status was documented. Enrolment was voluntary and costs for one examination per year were covered by the participants’ compulsory health insurance.

For the present analysis, we included participants aged 20 to 40 years attending a first examination between January 1988 and December 1993 for whom an additional examination was available eight to 12 years later. In case a participant attended multiple subsequent examinations within the baseline period we used the first one (t0). We choose the examination closest to 10 years following the baseline examination as 10 year follow-up examination (t1), if multiple subsequent examinations during the 8 to 12 year follow-up period were available.

Body Mass Index (BMI) was calculated from *measured body weight/(measured body height)* ^*2*^ (in kg/m^2^). BMI classification according to the World Health Organization was applied with underweight: BMI < 18.5 kg/m^2^, normal weight: 18.5 kg/m^2^ ≤ BMI < 25.0 kg/m^2^, overweight: 25 kg/m^2^ ≤ BMI < 30 kg/m^2^, obesity: BMI ≥ 30 kg/m^2^. Mean arterial pressure was calculated as 2/3 BP_Dias_ + 1/3 BP_Sys_. IFG was defined according to the cut-off of the American Diabetes Association (ADA) as FG ≥ 5.6 mmol/L (100 mg/dL). Individuals with FG>7.0 were included in the IFG category.

Ethical approval was obtained by the Vorarlberg ethic commission (EK-Nr. 2006-6/2).

### Statistical methods

#### Linear associations

We modeled the risks for the development or persistence of IFG associated with baseline BMI and BMI change using Poisson models with robust variance estimation. In addition an interaction term of BMI and BMI change was included to check if the relative risk associated with a specific BMI change differs by baseline BMI.

#### Categorical analyses

Poisson models were used to quantify the risk for the development or persistence of IFG associated with no change in BMI category, an upward change in BMI category or a downward change in BMI category. Out of 16 possible combinations of t0 and t1 BMI categories, those with at least one IFG case were included in the models (13 for men, 14 for women and development of IFG; 9 for men, 11 for women and persistence of IFG). However, we only report risks for those staying in their baseline category and risks associated with change from one category to the next lower or upper category. Robust variance estimation was used to attain nominal coverage of confidence intervals (95%).

#### Flexible models of baseline BMI and BMI change

We applied generalized additive models (GAM) using maximum likelihood, Poisson distribution and Pearson scaling in order to examine interactive and possible nonlinear associations of BMI and BMI change. GAM is an extension of the generalized linear model in which the outcome (here the risk) depends on flexible smooth functions of predictor variables (here BMI and BMI change) [18]. Colored contour plots were used to visualize the results of GAMs allowing to easily spot combinations of BMI and BMI change with about the same, or different level of risk for IFG.

All models were calculated separately for women and men and adjusted for age. Absolute risks are presented for 30 year old men and women. Thereto we estimated the linear predictor with age fixed at 30 years from Poisson models and GAMs respectively. The analyses of linear associations and those using categories of BMI were performed using SAS 9.3 (SAS Institute Inc., Cary, NC, USA.). Analyses with GAM using BMI and BMI change as continuous variables were performed with R (R Foundation for Statistical Computing) version 3.0.1 using the R package mgcv version 1.7–22.

## Results

Data from a total of 10,669 men and 14,261 women were included in the analyses. Characteristics of the study population, including metabolic parameters at baseline and at the follow-up examination are presented in Table 1. The median increase in BMI over ten years of follow-up was comparable in men and women (1.3 kg/m^2^). Like BMI all medians of all considered metabolic parameters except serum triglycerides increased over the 10 year period.

**Table 1:** Characteristics of the study population at baseline and 10-year follow-up.

	Men (N = 10,669)			Women (N = 14,261)		
	Baseline (t0)	Follow-up (t1)	Change (t1-t0)	Baseline (t0)	Follow-up (t1)	Change (t1-t0)
	Median (Q1, Q3)					
Age (years)	31.0 (26.6, 35.5)	41.1 (36.6, 45.5)	10.0 (9.3, 10.7)	30.3 (25.5, 35.1)	40.3 (35.5, 45.1)	10.0 (9.4, 10.6)
BMI (kg/m^2^)	24.1 (22.3, 26.1)	25.4 (23.4, 27.7)	1.3 (0.2, 2.5)	21.8 (20.0, 24.1)	23.0 (20.8, 26.2)	1.3 (0.0, 2.7)
MAP (mmHg)	93.3 (89.7, 100.0)	96.7 (90.0, 102.7)	1.7 (-6.3, 8.3)	90.0 (83.3, 95.0)	91.2 (83.3, 96.7)	1.7 (-6.7, 8.3)
FG (mmol/L)	4.5 (4.1, 5.0)	5.1 (4.6, 5.5)	0.5 (-0.1, 1.2)	4.4 (4.0, 4.9)	4.8 (4.4, 5.2)	0.4 (-0.2, 0.9)
TC (mmol/L)	5.2 (4.5, 5.9)	5.6 (4.9, 6.4)	0.4 (-0.1, 1.0)	4.9 (4.4, 5.6)	5.2 (4.7, 5.9)	0.4 (-0.2, 0.9)
TG (mmol/L)	1.2 (0.9, 1.8)	1.3 (0.9, 2.0)	0.0 (-0.4, 0.5)	1.0 (0.7, 1.3)	0.9 (0.7, 1.2)	-0.1 (-0.3, 0.2)
ɣ-GT (U/L)	23.3 (16.1, 35.8)	30.4 (19.7, 46.5)	3.6 (-3.6, 16.1)	14.3 (10.7, 19.7)	16.1 (12.5, 21.5)	0.0 (-3.6, 5.4)

*BMI* body mass index, *MAP* mean arterial pressure, *FG* fasting glucose, *TC* total cholesterol, *TG* triglycerides, *ɣ-GT* γ-glutamyltransferase

### Prevalence of overweight and obesity

The majority of participants were either normal weight or overweight at baseline (Table 2). More men than women were overweight at baseline (32.2% vs. 14.6%) and also at the follow-up visit (43.8% vs. 22.2%), whereas no significant gender difference was observed for obese subjects (baseline: 5.2% vs. 4.9%, follow-up: 11.0% vs. 10.4%). Most men and women stayed within their initial BMI category (69.2%), with the highest percentage (82.5%) in those being obese at baseline. Women being normal weight at baseline were more likely to stay normal weight than men (76.6% vs. 66.4%). Only 0.9% of obese men and 4.0% of obese women became normal weight during follow-up.

**Table 2:** Changes in BMI category over 10 years.

**Men**					
	**Follow-up (t1), N (%)** ^**a**^				
**Baseline (t0)**	**Underweight**	**Normal weight**	**Overweight**	**Obese**	**Total**
Underweight	16 (14.7)	88 (80.7)	4 (3.7)	1 (0.9)	109
Normal weight	17 (0.3)	4,366 (66.4)	2,122 (32.3)	67 (1.0)	6,572
Overweight	0 (0.0)	332 (9.7)	2,446 (71.2)	657 (19.1)	3,435
Obese	0 (0.0)	5 (0.9)	96 (17.4)	452 (81.7)	553
Total	33	4,791	4,668	1,177	10,669
**Women**					
	**Follow-up (t1), N (%)** ^**a**^				
**Baseline (t0)**	**Underweight**	**Normal weight**	**Overweight**	**Obese**	**Total**
Underweight	361 (31.7)	769 (67.5)	7 (0.6)	3 (0.3)	1,140
Normal weight	251 (2.4)	7,929 (76.6)	1,957 (18.9)	209 (2.0)	10,346
Overweight	0 (0.0)	280 (13.5)	1,113 (53.5)	688 (33.1)	2,081
Obese	0 (0.0)	28 (4.0)	89 (12.8)	577 (83.1)	694
Total	612	9,006	3,166	1,477	14,261

^a^ Given percentages sum up to 100 within rows

### Prevalence of IFG and Diabetes

The baseline prevalence of diabetes mellitus in men and women was low (1.3% and 0.9%) as was the diabetes prevalence at follow-up (1.9% and 1.1%). The prevalence of IFG was higher in men than in women at baseline (IFG: 10.3% vs. 7.7) as well as at follow-up (23.3% vs. 13.7%). IFG prevalence clearly increased with increasing baseline BMI category (Table 3).

**Table 3:** Baseline (t0) and 10-year follow-up (t1) prevalence of impaired fasting glucose and diabetes by baseline BMI category.

	Men				Women			
	Impaired fasting glucose		Diabetes mellitus		Impaired fasting glucose		Diabetes mellitus	
	(FG≤5.6 mmol/L)		(FG≤7.0 mmol/L)		(FG≤5.6 mmol/L)		(FG≤7.0 mmol/L)	
	t0	t1	t0	t1	t0	t1	t0	t1
	N (%)							
Underweight	9 (8.3)	22 (20.2)	1 (0.9)	0 (0.0)	64 (5.6)	105 (9.2)	8 (0.7)	3 (0.3)
Normal weight	581 (8.8)	1,280 (19.5)	80 (1.2)	83 (1.3)	713 (6.9)	1,201 (11.6)	73 (0.7)	65 (0.6)
Overweight	416 (12.1)	951 (27.7)	38 (1.1)	65 (1.9)	222 (10.7)	448 (21.5)	26 (1.3)	44 (2.1)
Obese	91 (16.5)	228 (41.2)	14 (2.5)	55 (10.0)	100 (14.4)	200 (28.8)	22 (3.2)	46 (6.6)
Total	1,097 (10.3)	2,481 (23.3)	133 (1.3)	203 (1.9)	1,099 (7.7)	1,954 (13.7)	129 (0.9)	158 (1.1)

### Risks for development and persistence of IFG by baseline BMI and BMI change

Linear models revealed an increased risk in men and women for development and persistence of IFG for each additional 1 kg/m^2^ at baseline and a further increase for each 1 kg/m^2^ gained over the 10 year follow-up (Table 4). For development of IFG BMI increase was more strongly associated with IFG risk than baseline BMI (RRs of 1.09 vs. 1.07, 1.06). However, included interaction terms of baseline BMI and BMI change indicated relative more importance of BMI change at lower levels of baseline BMI.

**Table 4:** Age adjusted linear associations of baseline BMI and BMI Change on relative risk for development and persistence of IFG.

	Development, RR (95%-CI)		Persistence, RR (95%-CI)	
	Men	Women	Men	Women
Baseline BMI [kg/m^2^]	1.07 (1.06, 1.08)	1.06 (1.05, 1.07)	1.07 (1.05, 1.09)	1.08 (1.06, 1.09)
BMI change [kg/m^2^]	1.09 (1.07, 1.11)	1.09 (1.08, 1.10)	1.04 (1.01, 1.08)	1.04 (1.01, 1.07)
	**Direction** ^**a**^ **/ p-value**		**Direction** ^**a**^ **/ p-value**	
Interaction (baseline BMI × BMI change)	neg. / 0.024	neg. / 0.022	neg. / 0.541	neg. / <0.001

^a^ neg.: BMI change has a weaker (multiplicative) effect on IFG risk when baseline BMI is higher. pos (not present).: BMI change has a stronger (multiplicative) effect on IFG risk when baseline BMI is higher

**Fig 1:**
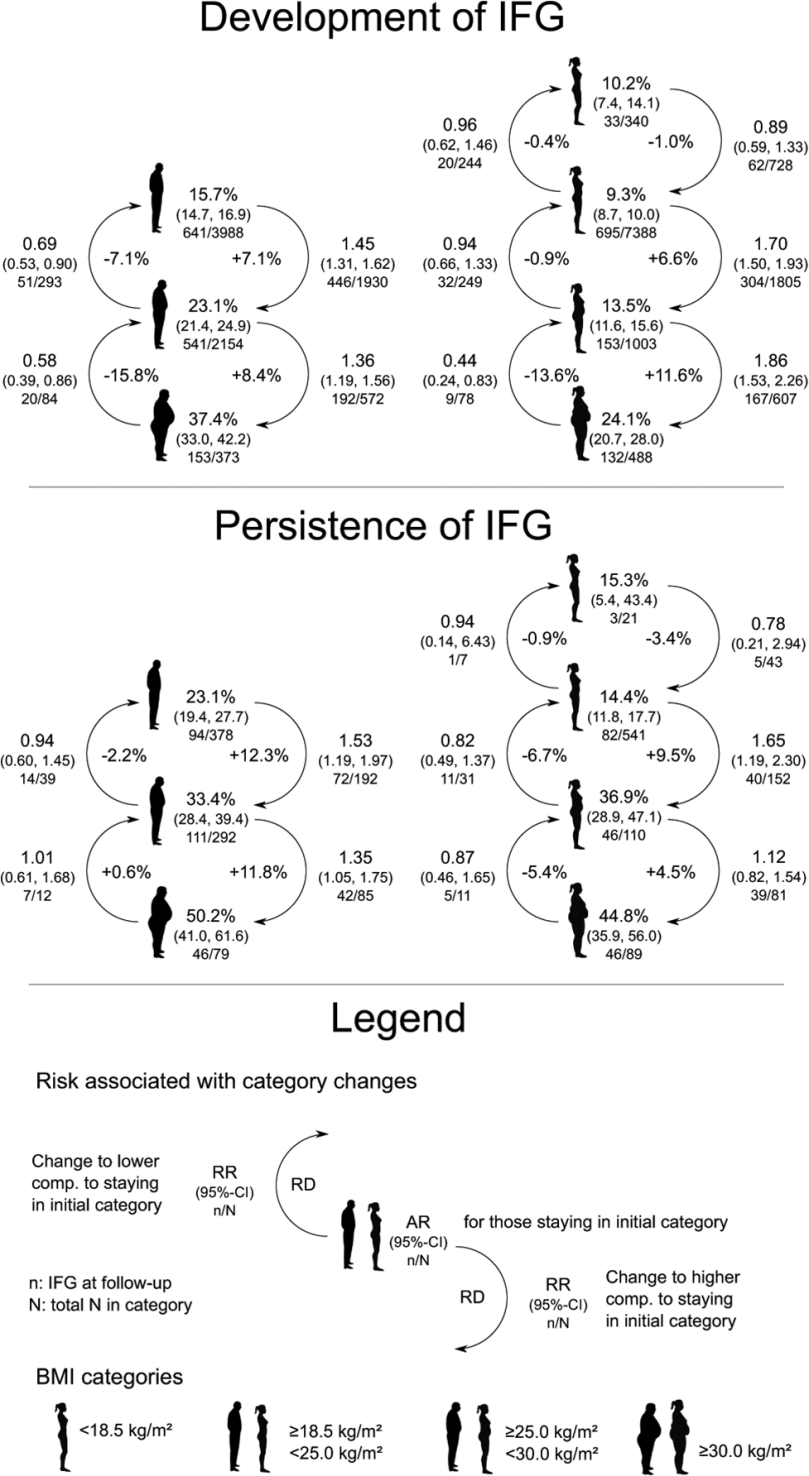
IFG risk by BMI category and category change. Absolute risk (AR) for development and persistence of impaired fasting glucose by BMI category, and relative risk (RR), risk difference (RD) associated with upward or downward change in BMI category for 30 year old women and men.

Risks for development and persistence of IFG for those staying in their initial BMI category and risks for participants changing BMI categories can be found in Fig 1. The risk for development of IFG after 10 years of follow-up was highest for men and women with persistent obesity (37.4% and 24.1%) and lowest in those with persistent normal weight (15.7%, 9.3%). Risks increased for participants changing into a higher BMI category and decreased for those transitioning to a lower category. Men transitioning from normal to overweight increased their risk of IFG by factor 1.45 (in absolute terms: from 15.7% by +7.1% to 22.8%), women by factor 1.71 (in absolute terms: from 9.3% by +6.6% to 15.9%), respectively. Men transitioning from overweight to normal weight reduced their risk of IGF by factor 0.69 (-7.1% absolute) compared to those staying overweight.

For IFG persistence the risk was increasing by baseline BMI category in men and women from 23.1% and 14.4% in stable normal weight to 50.2% and 44.8% in stable obesity. For the transition from normal to overweight the risk increased by factor 1.53 (from 23.1% by +12.3 to 35.4%) in men and by factor 1.66 (from 14.4% by +9.5% to 23.9%) in women.

**Fig 2:**
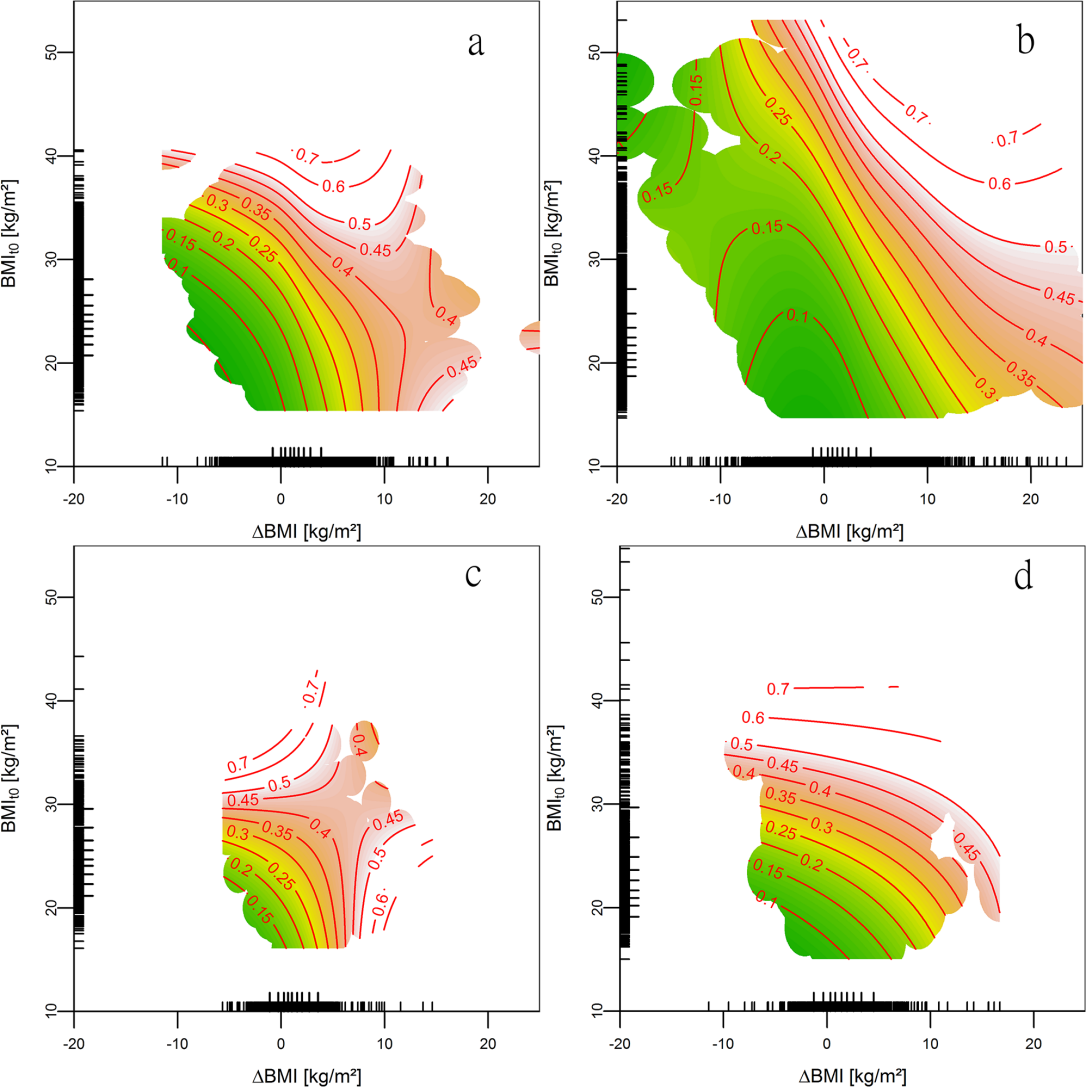
IFG risk by baseline BMI and BMI change estimated using generalized additive models. Absolute risk for development (a: men, b: women) and persistence (c: men, d: women) of impaired fasting glucose (IFG) by baseline BMI (y-axis) and ∆BMI (x-axis) for 30 year old individuals. Green indicates a low, yellow intermediate and red high risk. Contour lines (red lines) represent combinations of baseline BMI and BMI change being associated with the same risk. For example when looking at Fig 2a, at the “0.15” contour line (representing a 15% risk): There are men with a low baseline BMI and small BMI increase, and there are men with a high baseline BMI and pronounced weight decrease. Those men are at the same risk for development of IFG. Rather horizontal contour lines indicate baseline BMI is more strongly associated with IFG risk, rather vertical contour lines indicate BMI change is more strongly associated with IFG risk.

Flexible models using baseline BMI and BMI change as continuous variables confirmed the results of an association between baseline BMI, as well as BMI change with risks for development and persistence of IFG in men and women (Fig 2). For obese women with IFG, baseline BMI was a stronger predictor of IFG persistence than 10-year BMI change, indicated by the rather horizontal contour lines.

## Discussion

In this large cohort of young adults we found increasing median BMI over ten years in both genders. Impaired FG was more prevalent in men than in women. During 10 years of follow-up among men and women transitioning to a higher BMI category increased the risk of IFG whereas the transition to a lower BMI group reduced the risk compared to those with weight stability, especially in men. Excess weight at baseline was associated with higher risk for IFG persistence.

Overall, we found that weight stability was the most prevalent condition over 10 years, in particular in women. This finding is in line with other reports [19]. However, men typically stayed overweight and obese while women remained in the normal and overweight category. The et al. in a large representative cohort from the US observed that obese adolescents are at higher risk of developing severe obesity in adulthood [20]. In our study, only 9.5% of the initial overweight or obese participants were able to alter their body weight into normal weight. Adams et al. found that subjects from Health-AARP Diet and Health Study were lean until the age of 18 years, but gained considerable weight until the age of 50 years [2]. We found a higher risk of IFG persistence among participants with excess BMI. This suggests that additional programs targeting young adults may be needed to minimize potential complication of obesity in later life.

Our observation of a T2DM prevalence of 1.3 and 0.9% for men and women at baseline was somewhat lower than data among participants from the NHANES studies 2003–2006 (24–33 years of age: 4.4% total diabetes) and 2007–2009 (20–39 years of age: 2.5% total diabetes) [8,21]. In line with cross sectional studies using the current ADA cut point we have found an association between BMI and the prevalence of IFG [22,23]. However there is a lack of longitudinal studies on the risk of incident IFG. For incident T2DM a meta-analysis revealed a pooled relative risk of 1.87 (95%-CI: 1.67, 2.10) for one standard deviation increase of body mass index (BMI) [24].

Our study suggests that weight loss could reduce the onset of IFG in overweight and obese of both genders. This is consistent with findings for T2DM. Wannamethee et al. found weight gain to be associated with the onset of T2DM in men aged 40 to 59 years, while weight loss was associated with decreased risked for T2DM [25]. A recent meta-analysis revealed a linear relationship between BMI change and T2DM with a risk increase by 18% (95%-CI: 14, 22%) per 1kg/m^2^ increase [26]. However, in the Netherlands, no increased risk for T2DM was observed for weight gain over a 5-year period in a cohort aged 20–70 years [27]. Further evidence for a protective effect of weight reduction comes from an intervention study showing that lifestyle intervention reduced the 10-year T2DM risk by 34% (95%-CI: 24, 42%) [28].

Results of our continuous and flexible models suggest that decreasing BMI may be effective in reducing the risk for IFG development, especially in individuals who are not yet obese. For those having IFG already at baseline, baseline BMI was more strongly associated with IFG persistence than BMI change. Therefore weight control seems to be a reasonable approach for the prevention of IFG in the first place. But weight management may be less effective when IFG has already developed.

A major strength of our investigation is the large sample of adults that is significantly younger than in any other study to date. Height and weight were measured according to standard procedures. In addition, definition of IFG was based on current criteria for the diagnosis of IFG. Previous studies were based on the older cut point of 6.1 mmol/L and their reports on prevalence rates as well as risk estimates are therefore no more up to date [13,14].

Limitations of our study are a lack of information on some possible confounding variables, such as lifestyle, nutrition status, education-level [29], physical activity [30] and medication. Our study population probably includes a small group of people who may have been diagnosed earlier and, who thus may have received treatment. We therefore may underestimate the risk associated with weight gain. Study participation was voluntary and participants may be healthier than the general population, limiting generalizability. BMI provides little information on the distribution of body weight or body composition. Thus, other measures of body fat and (or) composition might be better predictors of incident IFG or IFG persistence. However, BMI is still the most widely used indicator of obesity in practice. Unfortunately, oral glucose tolerance tests were not performed. Therefore we could not compare our results to those that might be obtained when using impaired glucose tolerance to define prediabetes. The sample size for analysis concerning IFG persistence was comparatively low and risk estimates are less precise than those for development of IFG. Especially estimates for IFG persistence in some categories where numbers are particularly low should be treated with caution.

## Conclusion

In a large Austrian cohort, increasing mean body weight was observed in young adults over a 10-year follow-up. Increased weight at follow-up was associated with an increased risk of IFG, whereas decreased weight was associated with lower risks of IFG. Our results underline the need for the prevention of excess weight in young age in order to avoid long-term consequences of glycemic dysregulation.
